# The co-crystal *N*,*N*′-bis­[(pyridin-1-ium-2-yl)meth­yl]ethane­dithio­amide bis­(2,6-di­nitro­benzoate)–2,6-di­nitro­benzoic acid (1/4)

**DOI:** 10.1107/S1600536813023490

**Published:** 2013-09-04

**Authors:** Hadi D. Arman, Tyler Miller, Pavel Poplaukhin, Edward R. T. Tiekink

**Affiliations:** a Department of Chemistry, The University of Texas at San Antonio, One UTSA Circle, San Antonio, Texas 78249-0698, USA; b Chemical Abstracts Service, 2540 Olentangy River Rd, Columbus, Ohio 43202, USA; c Department of Chemistry, University of Malaya, 50603 Kuala Lumpur, Malaysia

## Abstract

The asymmetric unit of title co-crystal, C_14_H_16_N_4_S_2_^2+^·2C_7_H_3_N_2_O_6_^−^·4C_7_H_4_N_2_O_6_, comprises a centrosymmetric dipyridinium dication, a 2,6-di­nitro­benzoate anion and two independent 2,6-di­nitro­benzoic acid mol­ecules. The pyridin­ium rings are each approximately perpendicular to the central di­thio­amide unit [dihedral angle = 80.67 (12)°]. The carboxyl­ate/carb­oxy­lic acid groups are approximately perpendicular to the benzene ring to which they are attached [dihedral angles = 78.85 (16), 81.46 (19) and 71.28 (15)°]. By contrast, the major twist exhibited by a nitro group is manifested in a dihedral angle of 32.66 (17)°. The most prominent feature of the crystal packing is linear supra­molecular chains along [1-10], featuring O—H⋯O(carboxyl­ate) and pyridinium-N—H⋯O hydrogen bonds. These are consolidated into a three-dimensional architecture by thio­amide–nitro N—H⋯O, C—H⋯O and π–π [inter-centroid distance = 3.524 (2) Å] inter­actions. One of the nitro O atoms was refined over two sites; the major site was 0.65 (7) occupied.

## Related literature

For the 2:1 salts of 2,6-di­nitro­benzoate with isomeric *n*-({[(pyridin-1-ium-*n*-ylmeth­yl)carbamo­yl]formamido}­meth­yl)pyridin-1-ium, *n* = 2, 3 and 4, see: Arman *et al.* (2013 ▶). For co-crystals of 4-nitro­phenyl­acetic acid with *N*,*N*′-bis­(pyridin-3-ylmeth­yl)oxalamide and the thioxalamide analogue, see: Arman *et al.* (2012 ▶).
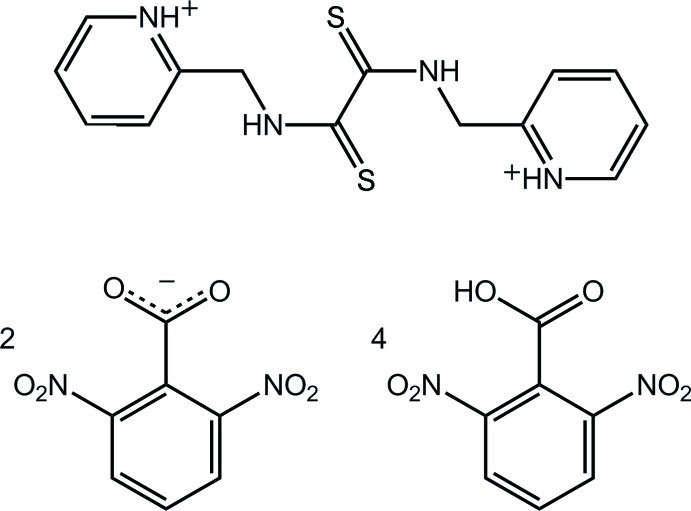


## Experimental

### 

#### Crystal data

C_14_H_16_N_4_S_2_^2+^·2C_7_H_3_N_2_O_6_^−^·4C_7_H_4_N_2_O_6_
*M* *_r_* = 1575.14
Triclinic, 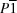

*a* = 11.157 (2) Å
*b* = 11.524 (3) Å
*c* = 14.967 (4) Å
α = 79.601 (18)°
β = 72.859 (17)°
γ = 61.237 (12)°
*V* = 1610.3 (7) Å^3^
*Z* = 1
Mo *K*α radiation
μ = 0.20 mm^−1^
*T* = 98 K
0.35 × 0.10 × 0.09 mm

#### Data collection

Rigaku AFC12/SATURN724 diffractometer
10659 measured reflections
7317 independent reflections
5680 reflections with *I* > 2σ(*I*)
*R*_int_ = 0.040
Standard reflections: 0

#### Refinement

*R*[*F* ^2^ > 2σ(*F* ^2^)] = 0.065
*wR*(*F* ^2^) = 0.166
*S* = 1.06
7317 reflections
518 parameters
4 restraints
H atoms treated by a mixture of independent and constrained refinement
Δρ_max_ = 1.01 e Å^−3^
Δρ_min_ = −0.50 e Å^−3^

### 

Data collection: *CrystalClear* (Molecular Structure Corporation & Rigaku, 2005 ▶); cell refinement: *CrystalClear*; data reduction: *CrystalClear*; program(s) used to solve structure: *SHELXS97* (Sheldrick, 2008 ▶); program(s) used to refine structure: *SHELXL97* (Sheldrick, 2008 ▶); molecular graphics: *ORTEPII* (Johnson, 1976 ▶) and *DIAMOND* (Brandenburg, 2006 ▶); software used to prepare material for publication: *publCIF* (Westrip, 2010 ▶).

## Supplementary Material

Crystal structure: contains datablock(s) general, I. DOI: 10.1107/S1600536813023490/xu5732sup1.cif

Structure factors: contains datablock(s) I. DOI: 10.1107/S1600536813023490/xu5732Isup2.hkl

Supplementary material file. DOI: 10.1107/S1600536813023490/xu5732Isup3.cml

Additional supplementary materials:  crystallographic information; 3D view; checkCIF report

## Figures and Tables

**Table 1:** Hydrogen-bond geometry (Å, °)

*D*—H⋯*A*	*D*—H	H⋯*A*	*D*⋯*A*	*D*—H⋯*A*
O2—H1o⋯O13^i^	0.84 (3)	1.70 (3)	2.536 (3)	169 (5)
O8—H2o⋯O14	0.85 (3)	1.70 (3)	2.546 (3)	178 (4)
N1—H1n⋯O14	0.88 (3)	1.86 (3)	2.733 (3)	171 (3)
N2—H2n⋯O15^ii^	0.88 (3)	2.53 (3)	3.202 (3)	134 (2)
C3—H3⋯O18^iii^	0.95	2.39	3.141 (5)	136
C12—H12⋯O16^iv^	0.95	2.41	3.301 (4)	157
C25—H25⋯O10^iv^	0.95	2.38	3.078 (5)	130

Symmetry codes: (i) 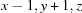; (ii) 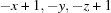; (iii) 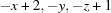; (iv) 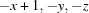.

**Fig. 1.**
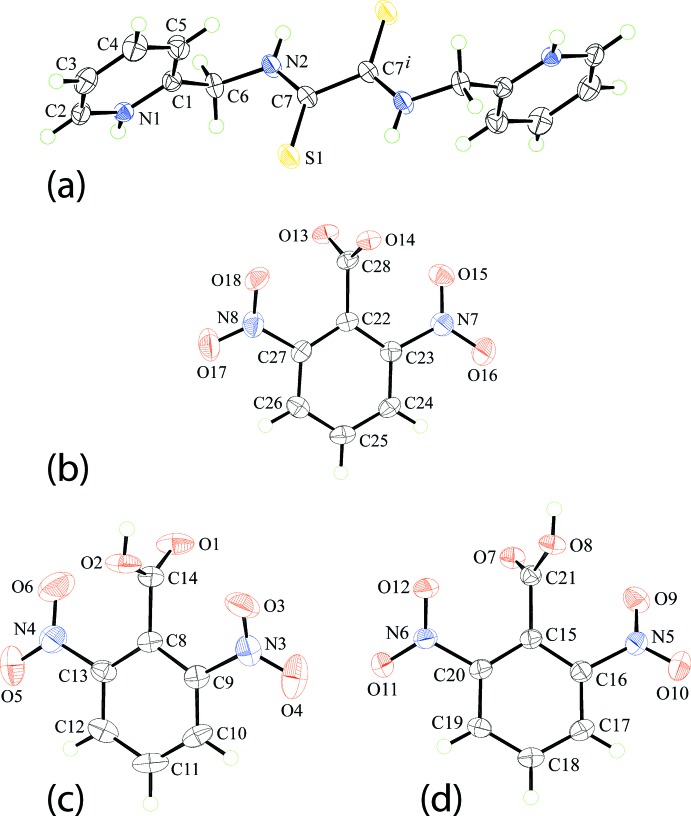
Molecular structures of the components of (I), showing atom-labelling scheme and displacement ellipsoids at the 50% probability level: (a) [C14H16N4S2]2+ (unlabelled atoms are related by the symmetry operation i: 1 - x, 1 - y, 1 - z), (b) 2,6-dinitrobenzoate anion (only the major component of the O17 atom is shown) and (c) the two independent 2,6-dinitrobenzoic acid molecules.

**Fig. 2.**
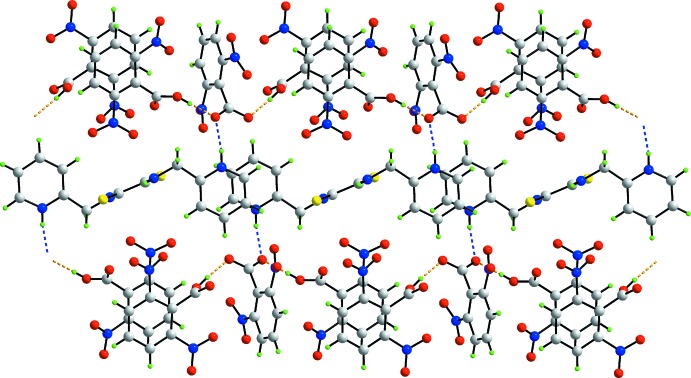
View of the supramolecular chain in (I). The O—H···O (orange) and N—H···O (blue) hydrogen bonds are shown as dashed lines.

**Fig. 3.**
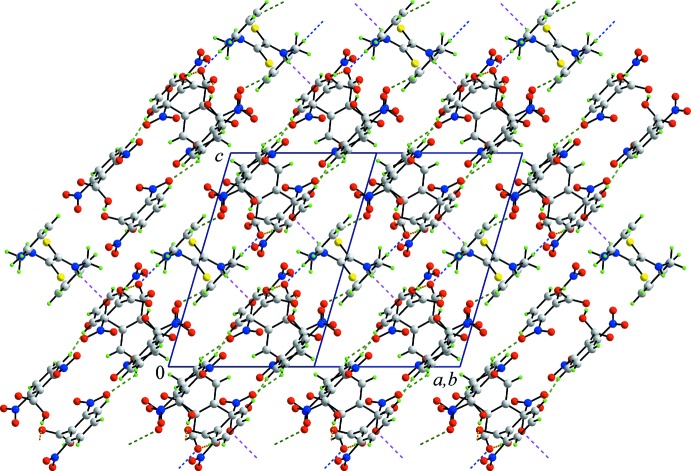
Unit-cell contents in (I) viewed down the axis of the supramolecular chain. The amide-N—H···O and C—H···O interactions are shown as pink and green dashed lines, respectively.

## supplementary crystallographic information

### 1. Comment

The title salt co-crystal (I) was isolated in continuation of on-going structural studies of salts/co-crystals formed between carboxylic acids, including 2,6-dinitrobenzoic acid (Arman *et al.*, 2013), and various pyridyl derivatives, such as the isomeric *N*,*N*'-bis(pyridin-n-ylmethyl)oxalamide series, where n = 2, 3 and 4, and their thioxalamide analogues (Arman *et al.*, 2012).

The asymmetric unit of (I) comprises half of a 2-({[(pyridin-1-ium-2- ylmethyl)carbamoyl]formamido}methyl)pyridin-1-ium dication, disposed about a centre of inversion, a 2,6-dinitrobenzoate anion and two molecules of 2,6-dinitrobenzoic acid, Fig. 1. The pyridin-1-ium rings lie to either side of the central dithioamide chromophore and adopt an almost perpendicular orientation forming a dihedral angle of 80.67 (12)°. In the anion, the carboxylate is inclined to the benzene ring to which it is attached forming a dihedral angle of 78.85 (16)°. A similar situation pertains in the neutral 2,6-dinitrobenzoic acid molecules where the comparable dihedral angles are 81.46 (19) and 71.28 (15)°. By contrast, while all nitro groups are twisted out of the plane of the benzene ring to which they are attached, the greatest twist is seen in the O12—N6—C20—C15 torsion angle of -32.7 (4)°.

The deprotonated carboxylate O13,O14 group is pivotal in the crystal packing, as each oxygen atom accepts a hydrogen bond from an adjacent molecule of 2,6-dinitrobenzoic acid, Table 1. As well, the O14 atom accepts a hydrogen bond from the pyridinium residue. A supramolecular chain results, base vector [1 - 1 0], as shown in Fig. 2. Chains are linked into a three-dimensional architecture by amide-N—H···O, C—H···O and π—π [inter-centroid distance between centrosymmetrically related C8–C13 rings = 3.524 (2) Å; symmetry operation = 1 - *x*, -*y*, 1 - *z*] contacts. Fig. 3 shows the unit-cell contents viewed down the axis of the chain.

### 2. Experimental

2,6-Dinitrobenzoic acid (Sigma-Aldrich, 0.1 mmol) was dissolved in methanol (5 ml) and added to this was a chloroform (10 ml) solution of *N*,*N*'-bis(pyridin-2-ylmethyl)thioxalamide (0.5 mmol). The mixture was heated and allowed to stand for slow evaporation affording red crystals.

### 3. Refinement

C-bound H-atoms were placed in calculated positions (C—H = 0.95–0.99 Å) and were included in the refinement in the riding model approximation with *U*_iso_(H) set to 1.2*U*_eq_(C). The O-and N-bound H-atoms were located in a difference Fourier map and were refined with a distance restraints of O—H = 0.84±0.01 Å and N—H = 0.88±0.01 Å, and with *U*_iso_(H) = 1.2*U*_eq_(N) and 1.5*U*_eq_(O). The maximum and minimum residual electron density peaks of 1.01 and 0.50 e Å^-3^, respectively, were located 1.25 Å and 0.79 Å from the O6 atom. One of the nitro-O atoms was refined over two sites; the major site was present 0.65 (7).

### Crystal data

**Table:**

C_14_H_16_N_4_S_2_^2+^·2C_7_H_3_N_2_O_6_^−^·4C_7_H_4_N_2_O_6_	*Z* = 1
*M**_r_* = 1575.14	*F*(000) = 806
Triclinic, *P*1	*D*_x_ = 1.624 Mg m^−^^3^
Hall symbol: -P 1	Mo *K*α radiation, λ = 0.71069 Å
*a* = 11.157 (2) Å	Cell parameters from 5825 reflections
*b* = 11.524 (3) Å	θ = 2.2–40.6°
*c* = 14.967 (4) Å	µ = 0.20 mm^−^^1^
α = 79.601 (18)°	*T* = 98 K
β = 72.859 (17)°	Block, red
γ = 61.237 (12)°	0.35 × 0.10 × 0.09 mm
*V* = 1610.3 (7) Å^3^	

### Data collection

**Table:**

Rigaku AFC12K/SATURN724 diffractometer	5680 reflections with *I* > 2σ(*I*)
Radiation source: fine-focus sealed tube	*R*_int_ = 0.040
Graphite monochromator	θ_max_ = 27.5°, θ_min_ = 2.0°
ω scans	*h* = −11→14
10659 measured reflections	*k* = −14→14
7317 independent reflections	*l* = −19→19

### Refinement

**Table:**

Refinement on *F*^2^	Primary atom site location: structure-invariant direct methods
Least-squares matrix: full	Secondary atom site location: difference Fourier map
*R*[*F*^2^ > 2σ(*F*^2^)] = 0.065	Hydrogen site location: inferred from neighbouring sites
*wR*(*F*^2^) = 0.166	H atoms treated by a mixture of independent and constrained refinement
*S* = 1.06	*w* = 1/[σ^2^(*F*_o_^2^) + (0.0657*P*)^2^ + 1.361*P*] where *P* = (*F*_o_^2^ + 2*F*_c_^2^)/3
7317 reflections	(Δ/σ)_max_ < 0.001
518 parameters	Δρ_max_ = 1.01 e Å^−^^3^
4 restraints	Δρ_min_ = −0.50 e Å^−^^3^

### Special details

**Table:**

Geometry. All s.u.'s (except the s.u. in the dihedral angle between two l.s. planes) are estimated using the full covariance matrix. The cell s.u.'s are taken into account individually in the estimation of s.u.'s in distances, angles and torsion angles; correlations between s.u.'s in cell parameters are only used when they are defined by crystal symmetry. An approximate (isotropic) treatment of cell s.u.'s is used for estimating s.u.'s involving l.s. planes.
Refinement. Refinement of *F*^2^ against ALL reflections. The weighted *R*-factor *wR* and goodness of fit *S* are based on *F*^2^, conventional *R*-factors *R* are based on *F*, with *F* set to zero for negative *F*^2^. The threshold expression of *F*^2^ > 2σ(*F*^2^) is used only for calculating *R*-factors(gt) *etc*. and is not relevant to the choice of reflections for refinement. *R*-factors based on *F*^2^ are statistically about twice as large as those based on *F*, and *R*- factors based on ALL data will be even larger.

### Fractional atomic coordinates and isotropic or equivalent isotropic displacement parameters (Å^2^)

**Table:**

	*x*	*y*	*z*	*U*_iso_*/*U*_eq_	Occ. (<1)
S1	0.70660 (8)	0.37681 (7)	0.41826 (6)	0.03648 (19)	
O1	0.1913 (3)	0.7776 (2)	0.13753 (16)	0.0472 (6)	
O2	0.0893 (3)	0.6553 (2)	0.22582 (16)	0.0450 (6)	
H1O	0.036 (4)	0.717 (3)	0.263 (2)	0.067*	
O3	0.3630 (3)	0.5734 (3)	0.2630 (2)	0.0622 (8)	
O4	0.5840 (3)	0.4611 (3)	0.19541 (18)	0.0619 (8)	
O5	0.1081 (3)	0.5406 (3)	−0.0511 (2)	0.0651 (8)	
O6	0.0553 (3)	0.6949 (3)	0.0351 (2)	0.0763 (9)	
O7	0.4776 (2)	0.26021 (19)	0.29655 (13)	0.0301 (4)	
O8	0.4287 (2)	0.08980 (19)	0.35613 (14)	0.0298 (4)	
H2O	0.5105 (18)	0.047 (3)	0.366 (2)	0.045*	
O9	0.4564 (2)	0.0995 (2)	0.16249 (15)	0.0417 (5)	
O10	0.2965 (3)	0.0709 (3)	0.1314 (2)	0.0676 (9)	
O11	0.0990 (2)	0.5488 (2)	0.44050 (14)	0.0351 (5)	
O12	0.2474 (2)	0.3455 (2)	0.47019 (14)	0.0387 (5)	
O13	0.90607 (19)	−0.17032 (19)	0.34152 (13)	0.0303 (4)	
O14	0.67500 (19)	−0.04280 (18)	0.38458 (12)	0.0267 (4)	
O15	0.7209 (2)	−0.2732 (2)	0.30113 (14)	0.0364 (5)	
O16	0.6182 (3)	−0.2497 (3)	0.19193 (18)	0.0653 (8)	
O17	0.952 (2)	0.139 (2)	0.1598 (9)	0.057 (3)	0.65 (7)
O17A	0.881 (9)	0.196 (6)	0.1569 (13)	0.073 (13)	0.35 (7)
O18	0.8389 (2)	0.1022 (2)	0.29373 (14)	0.0386 (5)	
N1	0.7670 (2)	0.0044 (2)	0.51789 (14)	0.0221 (4)	
H1N	0.744 (3)	−0.020 (3)	0.4758 (16)	0.027*	
N2	0.4912 (2)	0.3477 (2)	0.53239 (16)	0.0273 (5)	
H2N	0.4095 (18)	0.379 (3)	0.5734 (17)	0.033*	
N3	0.4577 (3)	0.5049 (3)	0.20045 (19)	0.0414 (6)	
N4	0.1306 (3)	0.5836 (3)	0.0067 (2)	0.0450 (7)	
N5	0.3340 (3)	0.1284 (3)	0.16974 (16)	0.0353 (5)	
N6	0.1660 (2)	0.4306 (2)	0.42466 (16)	0.0298 (5)	
N7	0.6920 (3)	−0.2256 (2)	0.22561 (17)	0.0356 (6)	
N8	0.8680 (3)	0.0999 (3)	0.20945 (19)	0.0383 (6)	
C1	0.6818 (3)	0.1241 (3)	0.55403 (17)	0.0238 (5)	
C2	0.8845 (3)	−0.0856 (3)	0.54572 (18)	0.0272 (5)	
H2	0.9417	−0.1685	0.5172	0.033*	
C3	0.9197 (3)	−0.0553 (3)	0.6158 (2)	0.0333 (6)	
H3	1.0020	−0.1169	0.6365	0.040*	
C4	0.8332 (3)	0.0669 (3)	0.6560 (2)	0.0362 (7)	
H4	0.8559	0.0886	0.7051	0.043*	
C5	0.7144 (3)	0.1571 (3)	0.62510 (19)	0.0321 (6)	
H5	0.6557	0.2408	0.6524	0.039*	
C6	0.5559 (3)	0.2091 (2)	0.51299 (19)	0.0265 (5)	
H6A	0.5858	0.2003	0.4443	0.032*	
H6B	0.4849	0.1759	0.5385	0.032*	
C7	0.5477 (3)	0.4270 (2)	0.48907 (18)	0.0246 (5)	
C8	0.2867 (3)	0.5484 (3)	0.10827 (18)	0.0274 (5)	
C9	0.4185 (3)	0.4676 (3)	0.12775 (18)	0.0297 (6)	
C10	0.5164 (3)	0.3527 (3)	0.0827 (2)	0.0370 (7)	
H10	0.6047	0.3013	0.0985	0.044*	
C11	0.4855 (4)	0.3125 (3)	0.0145 (2)	0.0415 (7)	
H11	0.5518	0.2327	−0.0162	0.050*	
C12	0.3575 (4)	0.3891 (3)	−0.0088 (2)	0.0368 (7)	
H12	0.3355	0.3631	−0.0561	0.044*	
C13	0.2618 (3)	0.5043 (3)	0.03774 (19)	0.0307 (6)	
C14	0.1825 (3)	0.6752 (3)	0.15902 (19)	0.0302 (6)	
C15	0.2550 (3)	0.2777 (3)	0.29713 (17)	0.0247 (5)	
C16	0.2231 (3)	0.2407 (3)	0.22679 (18)	0.0277 (5)	
C17	0.0909 (3)	0.3051 (3)	0.20708 (19)	0.0318 (6)	
H17	0.0738	0.2765	0.1585	0.038*	
C18	−0.0145 (3)	0.4107 (3)	0.2588 (2)	0.0312 (6)	
H18	−0.1053	0.4551	0.2461	0.037*	
C19	0.0110 (3)	0.4528 (3)	0.32935 (19)	0.0286 (5)	
H19	−0.0613	0.5262	0.3649	0.034*	
C20	0.1443 (3)	0.3856 (3)	0.34695 (18)	0.0266 (5)	
C21	0.4005 (3)	0.2085 (3)	0.31645 (17)	0.0246 (5)	
C22	0.7842 (3)	−0.0671 (2)	0.22087 (17)	0.0238 (5)	
C23	0.7426 (3)	−0.1301 (3)	0.17386 (18)	0.0271 (5)	
C24	0.7446 (3)	−0.1059 (3)	0.07891 (19)	0.0301 (6)	
H24	0.7145	−0.1507	0.0498	0.036*	
C25	0.7905 (3)	−0.0164 (3)	0.02744 (18)	0.0314 (6)	
H25	0.7936	−0.0002	−0.0375	0.038*	
C26	0.8318 (3)	0.0494 (3)	0.07111 (19)	0.0306 (6)	
H26	0.8633	0.1114	0.0366	0.037*	
C27	0.8268 (3)	0.0237 (3)	0.16622 (19)	0.0273 (5)	
C28	0.7888 (3)	−0.0975 (3)	0.32410 (17)	0.0254 (5)	

### Atomic displacement parameters (Å^2^)

**Table:**

	*U*^11^	*U*^22^	*U*^33^	*U*^12^	*U*^13^	*U*^23^
S1	0.0282 (4)	0.0248 (4)	0.0446 (4)	−0.0084 (3)	0.0039 (3)	−0.0065 (3)
O1	0.0520 (14)	0.0229 (11)	0.0448 (13)	−0.0102 (10)	0.0058 (11)	−0.0005 (9)
O2	0.0467 (13)	0.0259 (11)	0.0455 (13)	−0.0157 (10)	0.0170 (10)	−0.0123 (9)
O3	0.079 (2)	0.0403 (15)	0.0657 (17)	−0.0112 (14)	−0.0344 (16)	−0.0180 (13)
O4	0.0589 (17)	0.092 (2)	0.0520 (15)	−0.0505 (17)	−0.0298 (13)	0.0319 (14)
O5	0.0656 (18)	0.087 (2)	0.0663 (17)	−0.0437 (17)	−0.0345 (15)	−0.0006 (15)
O6	0.0590 (18)	0.0609 (19)	0.086 (2)	0.0067 (15)	−0.0384 (17)	−0.0163 (16)
O7	0.0281 (10)	0.0300 (10)	0.0325 (10)	−0.0137 (9)	−0.0095 (8)	0.0035 (8)
O8	0.0247 (9)	0.0256 (10)	0.0364 (10)	−0.0104 (8)	−0.0108 (8)	0.0077 (8)
O9	0.0303 (11)	0.0413 (13)	0.0436 (12)	−0.0095 (10)	−0.0037 (9)	−0.0080 (10)
O10	0.0554 (16)	0.072 (2)	0.0743 (19)	−0.0090 (14)	−0.0276 (14)	−0.0419 (15)
O11	0.0370 (11)	0.0287 (11)	0.0371 (11)	−0.0110 (9)	−0.0098 (9)	−0.0059 (8)
O12	0.0336 (11)	0.0412 (12)	0.0335 (11)	−0.0052 (10)	−0.0173 (9)	−0.0036 (9)
O13	0.0252 (9)	0.0296 (10)	0.0263 (9)	−0.0014 (8)	−0.0097 (8)	−0.0058 (7)
O14	0.0241 (9)	0.0238 (9)	0.0235 (9)	−0.0024 (8)	−0.0056 (7)	−0.0065 (7)
O15	0.0447 (12)	0.0299 (11)	0.0317 (10)	−0.0162 (10)	−0.0074 (9)	0.0004 (8)
O16	0.105 (2)	0.086 (2)	0.0476 (14)	−0.076 (2)	−0.0205 (15)	−0.0017 (14)
O17	0.066 (7)	0.067 (7)	0.056 (3)	−0.052 (6)	0.002 (3)	−0.013 (4)
O17A	0.14 (3)	0.09 (2)	0.046 (5)	−0.09 (3)	−0.022 (10)	0.006 (7)
O18	0.0471 (13)	0.0406 (12)	0.0364 (11)	−0.0217 (11)	−0.0145 (10)	−0.0081 (9)
N1	0.0242 (10)	0.0232 (11)	0.0206 (10)	−0.0118 (9)	−0.0059 (8)	−0.0010 (8)
N2	0.0224 (11)	0.0207 (11)	0.0346 (12)	−0.0075 (9)	−0.0024 (9)	−0.0055 (9)
N3	0.0518 (17)	0.0423 (16)	0.0402 (14)	−0.0278 (14)	−0.0217 (13)	0.0115 (12)
N4	0.0445 (16)	0.0393 (16)	0.0494 (16)	−0.0133 (13)	−0.0218 (13)	0.0019 (12)
N5	0.0390 (14)	0.0354 (14)	0.0277 (12)	−0.0107 (11)	−0.0120 (11)	−0.0047 (10)
N6	0.0268 (11)	0.0332 (13)	0.0269 (11)	−0.0109 (10)	−0.0082 (9)	−0.0009 (9)
N7	0.0483 (15)	0.0321 (13)	0.0311 (12)	−0.0224 (12)	−0.0051 (11)	−0.0077 (10)
N8	0.0400 (14)	0.0397 (15)	0.0414 (14)	−0.0237 (13)	−0.0045 (12)	−0.0084 (11)
C1	0.0251 (12)	0.0218 (12)	0.0251 (12)	−0.0119 (11)	−0.0052 (10)	−0.0003 (9)
C2	0.0274 (13)	0.0228 (13)	0.0287 (13)	−0.0102 (11)	−0.0088 (11)	0.0044 (10)
C3	0.0374 (15)	0.0340 (16)	0.0343 (15)	−0.0186 (13)	−0.0174 (13)	0.0068 (12)
C4	0.0491 (18)	0.0365 (16)	0.0347 (15)	−0.0229 (15)	−0.0219 (14)	0.0011 (12)
C5	0.0388 (15)	0.0274 (14)	0.0314 (14)	−0.0134 (13)	−0.0115 (12)	−0.0043 (11)
C6	0.0252 (12)	0.0187 (12)	0.0353 (14)	−0.0076 (11)	−0.0098 (11)	−0.0035 (10)
C7	0.0206 (12)	0.0217 (13)	0.0283 (12)	−0.0063 (11)	−0.0049 (10)	−0.0052 (10)
C8	0.0288 (13)	0.0237 (13)	0.0235 (12)	−0.0094 (11)	−0.0035 (10)	0.0004 (10)
C9	0.0331 (14)	0.0271 (14)	0.0260 (13)	−0.0127 (12)	−0.0080 (11)	0.0039 (10)
C10	0.0286 (14)	0.0274 (15)	0.0375 (15)	−0.0044 (12)	−0.0026 (12)	0.0048 (12)
C11	0.0446 (18)	0.0233 (14)	0.0327 (15)	−0.0052 (13)	0.0054 (13)	−0.0014 (11)
C12	0.0538 (19)	0.0298 (15)	0.0261 (13)	−0.0204 (14)	−0.0067 (13)	−0.0006 (11)
C13	0.0380 (15)	0.0246 (14)	0.0280 (13)	−0.0124 (12)	−0.0100 (12)	0.0005 (10)
C14	0.0336 (15)	0.0237 (14)	0.0287 (13)	−0.0101 (12)	−0.0059 (11)	−0.0015 (10)
C15	0.0248 (12)	0.0268 (13)	0.0227 (12)	−0.0117 (11)	−0.0079 (10)	0.0020 (10)
C16	0.0295 (13)	0.0274 (14)	0.0241 (12)	−0.0114 (11)	−0.0078 (10)	0.0007 (10)
C17	0.0350 (15)	0.0351 (15)	0.0297 (13)	−0.0172 (13)	−0.0148 (12)	0.0039 (11)
C18	0.0258 (13)	0.0334 (15)	0.0352 (14)	−0.0132 (12)	−0.0122 (11)	0.0040 (11)
C19	0.0237 (13)	0.0273 (14)	0.0294 (13)	−0.0076 (11)	−0.0077 (11)	0.0015 (10)
C20	0.0262 (13)	0.0290 (14)	0.0250 (12)	−0.0123 (11)	−0.0087 (10)	0.0016 (10)
C21	0.0246 (12)	0.0250 (13)	0.0223 (12)	−0.0098 (11)	−0.0061 (10)	−0.0001 (9)
C22	0.0209 (12)	0.0205 (12)	0.0232 (12)	−0.0026 (10)	−0.0057 (9)	−0.0049 (9)
C23	0.0291 (13)	0.0235 (13)	0.0266 (13)	−0.0096 (11)	−0.0061 (10)	−0.0044 (10)
C24	0.0319 (14)	0.0336 (15)	0.0272 (13)	−0.0137 (12)	−0.0087 (11)	−0.0079 (11)
C25	0.0282 (14)	0.0364 (16)	0.0224 (12)	−0.0077 (12)	−0.0072 (11)	−0.0037 (11)
C26	0.0260 (13)	0.0287 (14)	0.0298 (13)	−0.0094 (12)	−0.0037 (11)	0.0010 (11)
C27	0.0254 (13)	0.0246 (13)	0.0313 (13)	−0.0089 (11)	−0.0077 (11)	−0.0061 (10)
C28	0.0301 (13)	0.0220 (13)	0.0241 (12)	−0.0090 (11)	−0.0097 (10)	−0.0043 (9)

### Geometric parameters (Å, º)

**Table:**

S1—C7	1.651 (3)	C3—C4	1.390 (4)
O1—C14	1.209 (3)	C3—H3	0.9500
O2—C14	1.291 (3)	C4—C5	1.384 (4)
O2—H1O	0.845 (10)	C4—H4	0.9500
O3—N3	1.223 (4)	C5—H5	0.9500
O4—N3	1.230 (4)	C6—H6A	0.9900
O5—N4	1.209 (4)	C6—H6B	0.9900
O6—N4	1.217 (4)	C7—C7^i^	1.531 (5)
O7—C21	1.209 (3)	C8—C9	1.397 (4)
O8—C21	1.314 (3)	C8—C13	1.398 (4)
O8—H2O	0.846 (10)	C8—C14	1.520 (4)
O9—N5	1.214 (3)	C9—C10	1.377 (4)
O10—N5	1.221 (3)	C10—C11	1.381 (5)
O11—N6	1.226 (3)	C10—H10	0.9500
O12—N6	1.232 (3)	C11—C12	1.381 (5)
O13—C28	1.245 (3)	C11—H11	0.9500
O14—C28	1.260 (3)	C12—C13	1.386 (4)
O15—N7	1.226 (3)	C12—H12	0.9500
O16—N7	1.234 (3)	C15—C20	1.393 (4)
O17—O17A	0.75 (6)	C15—C16	1.395 (3)
O17—N8	1.227 (8)	C15—C21	1.517 (3)
O17A—N8	1.28 (2)	C16—C17	1.392 (4)
O18—N8	1.208 (3)	C17—C18	1.374 (4)
N1—C1	1.343 (3)	C17—H17	0.9500
N1—C2	1.350 (3)	C18—C19	1.389 (4)
N1—H1N	0.880 (10)	C18—H18	0.9500
N2—C7	1.323 (3)	C19—C20	1.389 (4)
N2—C6	1.446 (3)	C19—H19	0.9500
N2—H2N	0.879 (10)	C22—C27	1.390 (4)
N3—C9	1.480 (4)	C22—C23	1.391 (3)
N4—C13	1.467 (4)	C22—C28	1.532 (3)
N5—C16	1.473 (4)	C23—C24	1.394 (4)
N6—C20	1.478 (3)	C24—C25	1.381 (4)
N7—C23	1.471 (4)	C24—H24	0.9500
N8—C27	1.473 (3)	C25—C26	1.382 (4)
C1—C5	1.384 (4)	C25—H25	0.9500
C1—C6	1.509 (3)	C26—C27	1.391 (4)
C2—C3	1.373 (4)	C26—H26	0.9500
C2—H2	0.9500		
			
C14—O2—H1O	116 (3)	C8—C9—N3	119.4 (3)
C21—O8—H2O	114 (2)	C9—C10—C11	119.6 (3)
O17A—O17—N8	76.8 (15)	C9—C10—H10	120.2
O17—O17A—N8	68 (2)	C11—C10—H10	120.2
C1—N1—C2	123.9 (2)	C10—C11—C12	119.6 (3)
C1—N1—H1N	119 (2)	C10—C11—H11	120.2
C2—N1—H1N	117 (2)	C12—C11—H11	120.2
C7—N2—C6	122.7 (2)	C11—C12—C13	119.2 (3)
C7—N2—H2N	120 (2)	C11—C12—H12	120.4
C6—N2—H2N	117 (2)	C13—C12—H12	120.4
O3—N3—O4	124.4 (3)	C12—C13—C8	123.6 (3)
O3—N3—C9	118.1 (3)	C12—C13—N4	116.2 (3)
O4—N3—C9	117.5 (3)	C8—C13—N4	120.2 (3)
O5—N4—O6	123.0 (3)	O1—C14—O2	127.1 (3)
O5—N4—C13	119.1 (3)	O1—C14—C8	122.4 (3)
O6—N4—C13	117.6 (3)	O2—C14—C8	110.5 (2)
O9—N5—O10	123.9 (3)	C20—C15—C16	115.1 (2)
O9—N5—C16	118.7 (2)	C20—C15—C21	122.2 (2)
O10—N5—C16	117.4 (3)	C16—C15—C21	122.7 (2)
O11—N6—O12	125.0 (2)	C17—C16—C15	123.3 (3)
O11—N6—C20	117.7 (2)	C17—C16—N5	117.8 (2)
O12—N6—C20	117.2 (2)	C15—C16—N5	119.0 (2)
O15—N7—O16	123.6 (3)	C18—C17—C16	119.1 (2)
O15—N7—C23	118.5 (2)	C18—C17—H17	120.4
O16—N7—C23	117.9 (2)	C16—C17—H17	120.4
O18—N8—O17	121.3 (6)	C17—C18—C19	120.3 (3)
O18—N8—O17A	121.2 (13)	C17—C18—H18	119.9
O18—N8—C27	119.3 (2)	C19—C18—H18	119.9
O17—N8—C27	118.2 (5)	C18—C19—C20	118.8 (3)
O17A—N8—C27	114.0 (9)	C18—C19—H19	120.6
N1—C1—C5	118.4 (2)	C20—C19—H19	120.6
N1—C1—C6	115.3 (2)	C19—C20—C15	123.5 (2)
C5—C1—C6	126.3 (2)	C19—C20—N6	117.1 (2)
N1—C2—C3	119.0 (3)	C15—C20—N6	119.4 (2)
N1—C2—H2	120.5	O7—C21—O8	126.1 (2)
C3—C2—H2	120.5	O7—C21—C15	122.1 (2)
C2—C3—C4	118.9 (3)	O8—C21—C15	111.7 (2)
C2—C3—H3	120.5	C27—C22—C23	115.0 (2)
C4—C3—H3	120.5	C27—C22—C28	121.5 (2)
C5—C4—C3	120.5 (2)	C23—C22—C28	123.5 (2)
C5—C4—H4	119.7	C22—C23—C24	123.1 (3)
C3—C4—H4	119.7	C22—C23—N7	119.5 (2)
C1—C5—C4	119.3 (3)	C24—C23—N7	117.4 (2)
C1—C5—H5	120.4	C25—C24—C23	119.6 (2)
C4—C5—H5	120.4	C25—C24—H24	120.2
N2—C6—C1	113.4 (2)	C23—C24—H24	120.2
N2—C6—H6A	108.9	C24—C25—C26	119.5 (2)
C1—C6—H6A	108.9	C24—C25—H25	120.2
N2—C6—H6B	108.9	C26—C25—H25	120.2
C1—C6—H6B	108.9	C25—C26—C27	119.2 (3)
H6A—C6—H6B	107.7	C25—C26—H26	120.4
N2—C7—C7^i^	113.8 (3)	C27—C26—H26	120.4
N2—C7—S1	124.5 (2)	C22—C27—C26	123.7 (2)
C7^i^—C7—S1	121.7 (3)	C22—C27—N8	119.7 (2)
C9—C8—C13	114.5 (2)	C26—C27—N8	116.6 (2)
C9—C8—C14	121.7 (2)	O13—C28—O14	125.0 (2)
C13—C8—C14	123.8 (2)	O13—C28—C22	117.2 (2)
C10—C9—C8	123.5 (3)	O14—C28—C22	117.7 (2)
C10—C9—N3	117.1 (3)		
			
O17A—O17—N8—O18	−101 (2)	O9—N5—C16—C15	−24.1 (4)
O17A—O17—N8—C27	92.1 (16)	O10—N5—C16—C15	156.9 (3)
O17—O17A—N8—O18	101 (4)	C15—C16—C17—C18	0.1 (4)
O17—O17A—N8—C27	−105.4 (15)	N5—C16—C17—C18	−179.6 (2)
C2—N1—C1—C5	−1.1 (4)	C16—C17—C18—C19	0.3 (4)
C2—N1—C1—C6	−179.8 (2)	C17—C18—C19—C20	−0.5 (4)
C1—N1—C2—C3	0.8 (4)	C18—C19—C20—C15	0.3 (4)
N1—C2—C3—C4	0.2 (4)	C18—C19—C20—N6	−178.1 (2)
C2—C3—C4—C5	−0.7 (4)	C16—C15—C20—C19	0.1 (4)
N1—C1—C5—C4	0.5 (4)	C21—C15—C20—C19	178.4 (2)
C6—C1—C5—C4	179.0 (3)	C16—C15—C20—N6	178.4 (2)
C3—C4—C5—C1	0.4 (4)	C21—C15—C20—N6	−3.2 (4)
C7—N2—C6—C1	74.8 (3)	O11—N6—C20—C19	−32.3 (3)
N1—C1—C6—N2	−162.8 (2)	O12—N6—C20—C19	145.8 (3)
C5—C1—C6—N2	18.6 (4)	O11—N6—C20—C15	149.3 (2)
C6—N2—C7—C7^i^	172.3 (3)	O12—N6—C20—C15	−32.7 (4)
C6—N2—C7—S1	−8.2 (4)	C20—C15—C21—O7	−70.4 (3)
C13—C8—C9—C10	−0.8 (4)	C16—C15—C21—O7	107.8 (3)
C14—C8—C9—C10	−179.3 (3)	C20—C15—C21—O8	109.5 (3)
C13—C8—C9—N3	179.3 (2)	C16—C15—C21—O8	−72.2 (3)
C14—C8—C9—N3	0.7 (4)	C27—C22—C23—C24	0.7 (4)
O3—N3—C9—C10	−154.0 (3)	C28—C22—C23—C24	−177.1 (2)
O4—N3—C9—C10	23.7 (4)	C27—C22—C23—N7	−178.4 (2)
O3—N3—C9—C8	25.9 (4)	C28—C22—C23—N7	3.7 (4)
O4—N3—C9—C8	−156.4 (3)	O15—N7—C23—C22	−18.6 (4)
C8—C9—C10—C11	−0.2 (4)	O16—N7—C23—C22	158.8 (3)
N3—C9—C10—C11	179.7 (3)	O15—N7—C23—C24	162.2 (3)
C9—C10—C11—C12	1.0 (4)	O16—N7—C23—C24	−20.4 (4)
C10—C11—C12—C13	−0.8 (4)	C22—C23—C24—C25	0.5 (4)
C11—C12—C13—C8	−0.3 (4)	N7—C23—C24—C25	179.6 (3)
C11—C12—C13—N4	177.5 (3)	C23—C24—C25—C26	−1.0 (4)
C9—C8—C13—C12	1.0 (4)	C24—C25—C26—C27	0.2 (4)
C14—C8—C13—C12	179.5 (3)	C23—C22—C27—C26	−1.5 (4)
C9—C8—C13—N4	−176.7 (2)	C28—C22—C27—C26	176.4 (2)
C14—C8—C13—N4	1.9 (4)	C23—C22—C27—N8	177.7 (2)
O5—N4—C13—C12	5.7 (4)	C28—C22—C27—N8	−4.4 (4)
O6—N4—C13—C12	−168.2 (3)	C25—C26—C27—C22	1.1 (4)
O5—N4—C13—C8	−176.5 (3)	C25—C26—C27—N8	−178.2 (3)
O6—N4—C13—C8	9.6 (5)	O18—N8—C27—C22	−14.3 (4)
C9—C8—C14—O1	80.4 (4)	O17—N8—C27—C22	153.1 (16)
C13—C8—C14—O1	−98.1 (4)	O17A—N8—C27—C22	−168 (4)
C9—C8—C14—O2	−98.9 (3)	O18—N8—C27—C26	165.0 (3)
C13—C8—C14—O2	82.6 (3)	O17—N8—C27—C26	−27.6 (16)
C20—C15—C16—C17	−0.3 (4)	O17A—N8—C27—C26	11 (4)
C21—C15—C16—C17	−178.6 (2)	C27—C22—C28—O13	−76.6 (3)
C20—C15—C16—N5	179.4 (2)	C23—C22—C28—O13	101.1 (3)
C21—C15—C16—N5	1.1 (4)	C27—C22—C28—O14	100.7 (3)
O9—N5—C16—C17	155.6 (3)	C23—C22—C28—O14	−81.6 (3)
O10—N5—C16—C17	−23.4 (4)		

Symmetry code: (i) −*x*+1, −*y*+1, −*z*+1.

### Hydrogen-bond geometry (Å, º)

**Table:**

*D*—H···*A*	*D*—H	H···*A*	*D*···*A*	*D*—H···*A*
O2—H1o···O13^ii^	0.84 (3)	1.70 (3)	2.536 (3)	169 (5)
O8—H2o···O14	0.85 (3)	1.70 (3)	2.546 (3)	178 (4)
N1—H1n···O14	0.88 (3)	1.86 (3)	2.733 (3)	171 (3)
N2—H2n···O15^iii^	0.88 (3)	2.53 (3)	3.202 (3)	134 (2)
C3—H3···O18^iv^	0.95	2.39	3.141 (5)	136
C12—H12···O16^v^	0.95	2.41	3.301 (4)	157
C25—H25···O10^v^	0.95	2.38	3.078 (5)	130

Symmetry codes: (ii) *x*−1, *y*+1, *z*; (iii) −*x*+1, −*y*, −*z*+1; (iv) −*x*+2, −*y*, −*z*+1; (v) −*x*+1, −*y*, −*z*.
